# The Ras–PI3K Signaling Pathway Is Involved in Clathrin-Independent Endocytosis and the Internalization of Influenza Viruses

**DOI:** 10.1371/journal.pone.0016324

**Published:** 2011-01-20

**Authors:** Yoichiro Fujioka, Masumi Tsuda, Tomoe Hattori, Junko Sasaki, Takehiko Sasaki, Tadaaki Miyazaki, Yusuke Ohba

**Affiliations:** 1 Laboratory of Pathophysiology and Signal Transduction, Hokkaido University Graduate School of Medicine, Sapporo, Japan; 2 Department of Bioresources, Hokkaido University Research Center for Zoonosis Control, Sapporo, Japan; 3 Division of Microbiology, Department of Pathology and Immunology, Akita University School of Medicine, Akita, Japan; Hallym University, Republic of Korea

## Abstract

**Background:**

Influenza virus infection causes highly contagious, severe respiratory disorders and gives rise to thousands of deaths every year; however, the efficacy of currently approved defense strategies, including vaccines and neuraminidase inhibitors, is limited because the virus frequently acquires resistance via antigen drift and reassortment. It is therefore important to establish a novel, effective therapeutic strategy that is effective irrespective of viral subtype.

**Methodology/Principal Findings:**

Here, we identify the Ras–phosphoinositide 3-kinase (PI3K) signaling pathway as a host-cell regulatory mechanism for influenza virus entry. The binding of Ras to PI3K is specifically involved in clathrin-independent endocytosis, endosomal maturation, and intracellular transport of viruses, which result in decreased infectious efficacy of different subtypes of influenza viruses in cells lacking the Ras–PI3K interaction. Moreover, influenza virus infection indeed triggered Ras activation and subsequent PI3K activation in early endosomes.

**Conclusions/Significance:**

Taken together, these results demonstrate that the Ras–PI3K signaling axis acts as a host-oriented mechanism for viral internalization. Given that virus incorporation is a process conserved among virus subtypes and species, this signaling pathway may provide a target for potent, well-tolerated prophylactics and therapeutics against a broad range of viruses.

## Introduction

Influenza spreads around the world in seasonal epidemics, resulting in the death of hundreds of thousands of people annually—millions in pandemic years. Several worldwide influenza outbreaks that arose in the last century claimed the lives of tens of millions; each of these pandemics was caused by the introduction of a viral strain with an HA subtype not found in other human influenza viruses. In February–April 2009, a novel H1N1 strain—a reassortant of human and swine influenza viruses with Eurasian avian-like swine viruses—appeared in Mexico, followed by the United States and other nations. At present, neuraminidase (NA) inhibitors such as oseltamivir and zanamivir have been efficacious against the current H1N1 strain of swine origin; however, the virus may acquire resistance to the available antiviral drugs. In fact, the incidence rate of oseltamivir-resistant H1N1 influenza viruses in the United State increased from 0.7% in the 2006–2007 influenza season to 98.5% in the 2008–2009 influenza season [1]. Although vaccines are also available for the prevention and control of influenza virus infection, they need to be frequently revised—typically every 1–3 years in the case of seasonal influenza vaccines—to accommodate mutations in the HA and NA proteins of the circulating viruses (antigen drift). In addition, it has recently been reported that in response to variation in neutralizing antibody pressure between individuals, the influenza A virus evolves by adjusting receptor binding avidity via amino acid substitutions throughout the HA globular domain, many of which simultaneously alter antigenicity [2]. Host-oriented mechanisms involved in influenza infection, rather than viral proteins, should therefore be envisaged as promising targets for the development of novel, potent therapeutics that are effective irrespective of viral subtype.

Indeed, several genome-wide screenings succeed to identify host proteins that participate in every step of influenza virus infection [3], [4], [5], [6]. These include signaling molecules related to viral replication, innate immune responses, and apoptosis, as well as signal transduction pathways regulating more fundamental and physiological processes such as cell proliferation, differentiation, and survival. Among them, the family of lipid kinases phosphoinositide-3 kinases (PI3Ks), a key regulator in many cellular processes [7], is one of the commonly listed host factors across the literature [3], [4], [5], [6], indicating the crucial role of this protein in influenza virus infection. Accordingly, it was reported that the multifunctional viral non-structural protein (NS1) binds directly to the regulatory subunit p85β, but not p85α, and stimulates the lipid kinase activity of the p85-associated catalytic subunit p110 of PI3K [8], [9], [10], [11], [12]. Although this PI3K activation is apparently of importance in promoting efficient virus replication at a late step of the infection [11], the underlying mechanisms by which PI3K contributes to viral infection remain controversial. In fact, it was paradoxically reported that PI3K inhibition is effective only in the initial stage of infection [13], [14], indicating a bivalent role for PI3K in both early and late stages of infection.

The pathway of influenza transmission is a multistep process involving adsorption of viral HA to sialylated host surface proteins, entry of the virus into cells by endocytosis, and trafficking from early to late endosomes. The viruses then fuse with the membrane of late endosomes to release the viral genes necessary for replication [15], [16], [17]. A growing body of evidence shows that influenza viruses undergo clathrin-dependent endocytosis. However, the virus can still enter the cells even with inhibition of clathrin- or caveolae-mediated endocytosis, suggesting that influenza viruses infect cells through an additional clathrin- and caveolae-independent endocytic pathway [18]. We have recently reported that PI3K bound to Ras is preferentially translocated to the endosome and activated in this subcellular compartment; however, a role for this binding in the endosome has yet to be determined [19]. In this study, we demonstrate that Ras–PI3K signaling plays an indispensable role in the regulation of clathrin-independent endocytosis and influenza virus entry.

## Results

### Ras–PI3K interaction regulates clathrin-independent endocytosis

**Figure 1:**
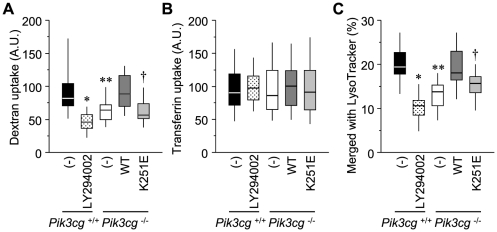
Requirement for the Ras–PI3K interaction in clathrin-independent endocytosis. (A–C) Cells described at the bottom were incubated with fluorescent dextran (A), transferrin (B), or dextran and LysoTracker Red (C). After extensive washing, the cells were observed under a fluorescence microscope, and the uptake of dextran (A) and transferrin (B) was determined by measuring the fluorescence intensities. In (C), colocalization of dextran and LysoTracker was quantified using image-processing software. The distributions of the values are displayed as box-and-whisker plots: the highest and lowest boundaries of the box represent the 25th and 75th percentiles, respectively; whiskers above and below the box designate the 5th and 95th percentiles, respectively; and the line within the box indicates the median. The values were obtained from at least 30 cells pooled from three separate experiments. *, *P* = 3.1×10^−16^; **, *P* = 6.2×10^−8^; †, *P* = 3.2×10^−8^ (A). *, *P* = 2.2×10^−9^; **, *P* = 2.8×10^−6^; †, *P* = 2.0×10^−4^ (C).

In order to test whether PI3K participates in the regulation of endocytosis, we first prepared mouse embryonic fibroblasts (MEFs) from wild-type mice and mice deficient in *Pik3cg* (the gene encoding the catalytic subunit of PI3Kγ, a subtype of PI3K). Clathrin-dependent and -independent endocytosis in these cells was assessed by uptake of fluorescently labeled transferrin and dextran, respectively. The treatment of wild-type MEFs with the pan-PI3K inhibitor LY294002 significantly reduced the uptake of dextran, but not that of transferrin (Figure 1A, B), confirming that PI3K is specifically involved in clathrin-independent endocytosis, as reported previously [20]. *Pik3cg*-deficiency in MEFs also displayed an inhibitory effect on dextran uptake; however, the inhibitory effect observed in the mutant MEFs was significantly less than that of LY294002-treated cells (Figure 1A, *P* = 1.2×10^−7^, LY294002-treated *Pik3cg* ^+/+^ vs. *Pik3cg* ^−/−^). Because PI3Kα and PI3Kβ are expressed in these mutant cells [21], the difference in capacity for dextran uptake between LY294002-treated wild-type and *Pik3cg*-deficient MEFs could be accounted for by the remaining activity of the α and β subtypes of PI3K in the *Pik3cg* ^−/−^ cells. Interestingly, the PI3Kγ K251E mutant, which fails to bind to Ras [22] and hence to translocate to the endosome via activated Ras (Figure S1A, B), was unable to display a compensatory effect, unlike its wild-type counterpart (Figure 1A), while the expression levels were shown to be comparable by immunoblotting (Figure S1C). Given that the mutants lacking Ras-binding retain the catalytic activity [23], [24], this result demonstrates the significance of Ras-mediated activation of PI3K in the regulation of clathrin-independent endocytosis.

**Figure 2 pone-0016324-g002:**
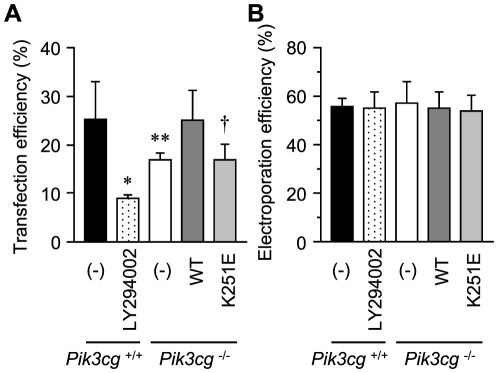
Impairment of events related to endocytosis in cells lacking Ras–PI3K binding. An expression vector for CFP was introduced into cells denoted at the bottom using the cationic lipid-mediated transfection method FuGene HD (A) or electroporation using the Amaxa nucleofection system (B). After 24 h, the gene transduction efficiency was calculated by counting the number of fluorescence-positive and -negative cells under a fluorescence microscope. The total cell number counted was at least 1000 in each experiment. Values are the mean ± S.D. of data pooled from three independent experiments. *, *P* = 8.7×10^−4^; **, *P* = 1.5×10^−3^; †, *P* = 4.1×10^−3^ (A).

The Ras–PI3K pathway also participates in endosome maturation. Simultaneous visualization of early endosomes and late endosomes/lysosomes by fluorescently labeled dextran and LysoTracker, respectively, revealed that dextran transfer into late endosomes was significantly delayed in cells in which PI3K activity or Ras–PI3Kγ binding was absent (Figure 1C, lanes 2,3, and 5; Figure S1D). Consistent with this finding, cellular events related to endosomal maturation were specifically disturbed in cells lacking the Ras–PI3Kγ interaction. The efficacy of transfection, in which lipid-conjugated plasmid DNA is thought to be transmitted into cells via fluid phase, clathrin-independent endocytosis [25], was significantly impaired in *Pik3cg*-deficient MEFs. This impairment was rescued by the expression of wild-type PI3Kγ but not by expression of the mutant PI3Kγ (Figure 2A). In contrast, gene transfer by electroporation was not affected by any of the tested conditions (Figure 2B), indicating that endocytosis-mediated events, but not protein expression, are specifically disrupted in *Pik3cg* ^−/−^ MEFs.

### Ras–PI3K signaling regulates influenza virus entry

**Figure 3:**
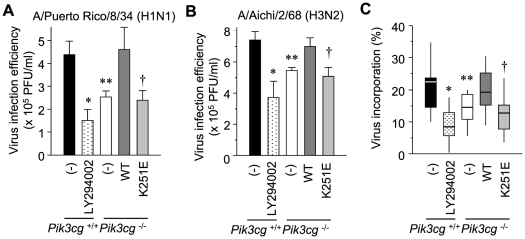
Impediment of influenza A virus infection in the absence of the Ras–PI3K interaction. (A and B) MEFs described at the bottom were infected with A/Puerto Rico/8/34 (H1N1, PR8; A) or A/Aichi/2/68 (H3N2, Aichi; B) at a multiplicity of infection (MOI) of 250 PFU/cell. The titers of infectious viral particles produced from these infections were determined using an MDCK plaque assay and are represented as PFU/ml. Values are expressed as the mean ± S.D. of data pooled from three independent experiments. *, *P* = 3.3×10^−8^; **, *P* = 5.8×10^−6^; †, *P* = 3.1×10^−6^ (A). *, *P* = 4.6×10^−3^; **, *P* = 2.6×10^−3^; †, *P* = 2.0×10^−3^ (B). (C) Cells indicated at the bottom were electroporated with expression vectors for CFP-Rab5 and YFP-Rab7. After 24 h the cells were infected with PR8 for 1 h, fixed, stained with an anti-NP antibody, and imaged using a confocal microscope. Percentages of virus particles localized in vesicles in the presence of Rab5 or Rab7 were determined using image processing software. These particles were assumed to be virus particles incorporated in cells. Values were obtained from at least 30 cells from three independent experiments and are presented in box-and-whisker plots. *, *P* = 1.9×10^−7^; **, *P* = 1.5×10^−3^; †, *P* = 3.6×10^−4^.

The fact that both endosome maturation and activation of PI3K are implicated in influenza virus infection, especially in the very early stages [13], [14], encouraged us to examine the status of influenza virus infection in our system. As expected, challenge with the A/Puerto Rico/8/34 (H1N1; PR8) strain resulted in reduced titers of progeny viruses in LY294002-treated and *Pik3cg*-deficient cells as assessed by a conventional plaque assay (Figure 3A). This resistance to influenza was restored by the expression of wild-type PI3Kγ but not by that of the K251E mutant (Figure 3A). Similar infection profiles were obtained when A/Aichi/2/68 (H3N2; Aichi) viruses were used (Figure 3B). We can therefore state that suppression of the Ras–PI3K interaction is effective in a manner independent of the influenza subtype.

To test whether the internalization and intracellular trafficking of infected viral particles are regulated by the Ras–PI3K interaction, we prepared the aforementioned cell lines expressing both CFP-Rab5 and YFP-Rab7, markers for early and late endosomes, respectively. Immunofluorescence analysis using an antibody against the influenza nucleoprotein NP revealed that the total number of adsorbed virus particles was comparable among the tested conditions (Figure S2A); however, the ratio of particles colocalized with Rab proteins to the total number of particles, which is expected to represent the virus entry rate, was reduced in LY294002-treated wild-type MEFs, *Pik3cg*-deficient MEFs, and knockout MEFs expressing the K251E mutant (Figure 3C and Figure S2B, C). These findings are consistent with the results of dextran uptake (see Figure 1A). The delivery of viral particles from early to late endosomes, which can be analyzed by calculating the ratio of virus particles colocalized with CFP-Rab5 and YFP-Rab7 to the total number of internalized particles, was concomitantly retarded in knockout cells and in mutant PI3Kγ-expressing cells (Figure S2C). This suggests that Ras–PI3K signaling also modulates virus trafficking from early to late endosomes via the regulation of endosomal maturation (see Figure 1C). The PI3K inhibitor promoted retention in early endosomes more efficiently than *Pik3cg* ablation (Figure S2C, *P* = 4.1×10^−3^), indicating that other subtypes of PI3Ks also play a role in viral trafficking.

### Ras–PI3K is required for influenza-dependent upregulation of endocytosis

**Figure 4:**
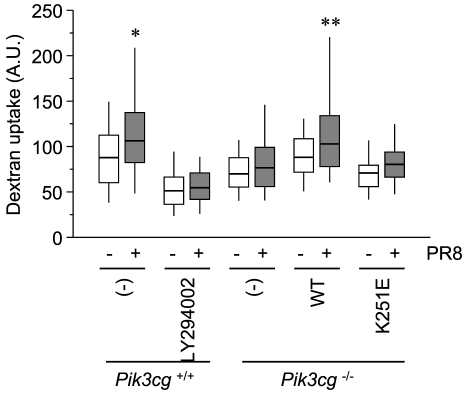
Influenza viruses promote clathrin-independent endocytosis via Ras–PI3K signaling. The cells denoted at the bottom were labeled with fluorescent dextran for 30 min in the presence or absence of PR8 at an MOI of 2.5. Dextran incorporation into the cells was determined as in Figure 1A. Values were obtained from at least 40 cells from three independent experiments and are presented in box-and-whisker plots. *, *P* = 4.9×10^−5^; **, *P* = 1.8×10^−3^.

Influenza viruses may also exploit the Ras–PI3K signaling pathway to expedite their incorporation into cells, in which they create more favorable conditions for themselves. As shown in Figure 4, dextran uptake was significantly increased upon PR8 infection in wild-type MEFs and *Pik3cg*-deficient MEFs expressing wild-type PI3K. In contrast, PI3K inhibitor-treated wild-type MEFs, knockout MEFs, and knockout MEFs expressing mutant PI3K displayed similar lower basal incorporation activities, with marginal upregulation after viral infection (Figure 4), thereby highlighting Ras–PI3K signaling as a critical host-oriented mechanism for the incorporation of influenza viruses.

### Influenza virus infection activates Ras–PI3K signaling in the endosomes

**Figure 5:**
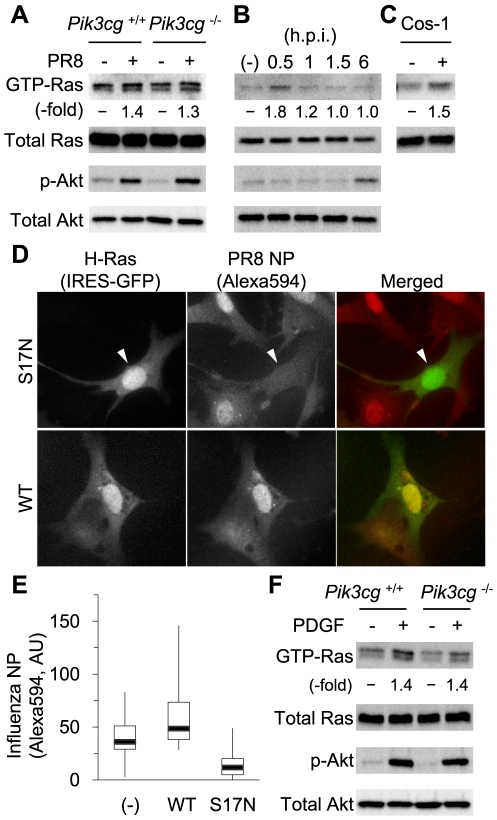
Activation of Ras–PI3K signaling by influenza infection. (A) Wild-type and *Pik3cg*-deficient MEFs were infected with PR8 or mock infected. After 0.5 h, the cells were subjected to a pull-down assay to determine Ras activity. Cells were also infected with PR8 for 6 h and subjected to immunoblotting to determine Akt phosphorylation. (B) Ras activity and Akt phosphorylation in wild-type MEFs at the indicated times were determined as in (A). (C) Cos-1 cells were infected with PR8 for 30 min or left untreated and subjected to a Bos pull-down assay. (D and E) MEFs expressing the dominant negative mutant (Ras S17N) or wild type (WT) of Ras were infected with PR8, stained with an anti-NP antibody, and observed by fluorescence microscopy. The arrowhead indicates a cell with fluorescence of EGFP bicistronically expressed with Ras S17N, in which influenza virus infection was inhibited (D). The fluorescence intensity of AlexaFluor594 (NP) for each cell was plotted and is presented in box-and-whisker plots (E). *, *P* = 0.027; **, *P* = 1.5×10^−3^ (F) Comparable Ras activation and Akt phosphorylation induced by a growth factor in mutant cells. Wild-type and *Pik3cg* ^−/−^ MEFs were stimulated by platelet-derived growth factor (PDGF) for 10 min, and Ras activity and Akt phosphorylation were determined by pull-down analysis and immunoblotting, respectively.

Next, we examined whether influenza infection does in fact activate Ras–PI3K signaling. To examine activity of endogenous Ras, we used the Bos pull-down method in which GTP-bound, active Ras is recovered from cell lysates by its preferential avidity to the GST-fused Ras-binding domain (RBD) of c-Raf1 [26]. As shown in Figure 5A, B, Ras activation was efficiently promoted at 30 minutes after PR8 infection, irrespective of the presence or absence of PI3Kγ. Ras activation by PR8 could also be observed in Cos-1 cells (Figure 5C). In addition, expression of the dominant negative mutant of Ras (Ras S17N) inhibited influenza virus infection, whereas expression of its wild-type counterpart rather enhanced the infection (Figure 5D, E), together demonstrating a requirement for Ras in influenza virus infection. We further assessed the phosphorylation status of Akt, a commonly used marker of PI3K activation, by immunoblotting using an antibody specific for phosphorylated Akt (p-Akt, S473). Surprisingly, although influenza infection was definitely inhibited in *Pik3cg*-deficient cells, Akt phosphorylation was induced by virus exposure for 6 hours (Figure 5A). Upon PDGF stimulation, essentially similar Ras and Akt activation was observed, even in the absence of PI3Kγ (Figure 5F), which is consistent with a previous report that the Akt phosphorylation level induced by extracellular stimuli is not altered by ablation of PI3Kγ [27]. Moreover, although the PI3K binding to Ras is critical for influenza virus infection (see Figure 3A, B), the kinetics of Akt phosphorylation were apparently distinct from those of Ras activity (Figure 5B). In fact, kinetics of Akt phosphorylation induced by influenza virus infection are reported to be in a manner dependent on cell contexts and viral strains [13]. We therefore postulated that PI3K activation is disturbed in a manner specific for subcellular localization and that in our system Akt phosphorylation at the whole-cell level is an inappropriate indicator of PI3K activity at such an early stage of infection.

**Figure 6:**
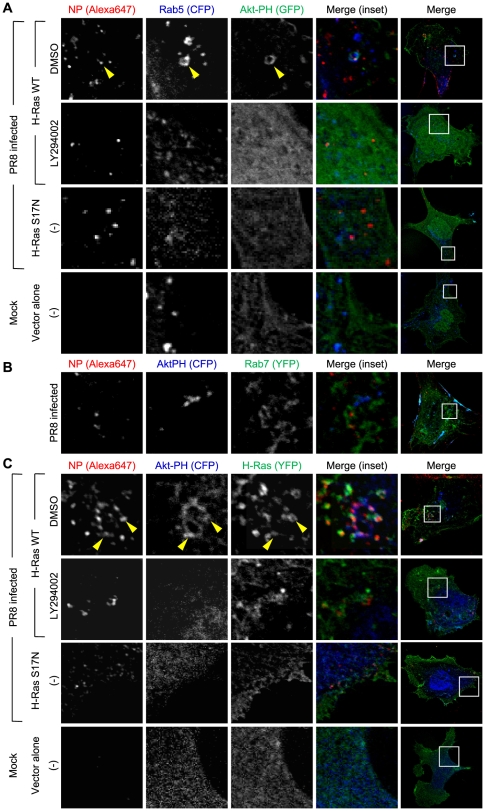
Colocalization of virus particles, Akt-PH, and Ras in early endosomes upon influenza infection. (A) MEFs expressing CFP-Rab5 and Akt-PH-GFP (and H-Ras S17N in the third panels) were mock-infected (Mock) or infected with PR8. After 30 min, the cells were fixed, stained with an anti-NP antibody and further incubated with AlexaFluor647-conjugated secondary antibodies. The cells were then observed using confocal microscopy. Representative images are shown. In the top panels, an arrowhead indicates colocalization of NP, Rab5, and Akt-PH. (B) MEFs expressing CFP-Akt-PH and YFP-Rab7 were processed as in (A). (C) MEFs expressing Akt-PH-CFP and YFP-H-Ras WT were mock-infected or infected with PR8. After 30 min, the cells were fixed and subjected to immunofluorescence as in (A). In the top panels, arrowheads indicate colocalization among NP, Akt-PH, and H-Ras.

The GFP-tagged pleckstrin homology (PH) domain of Akt (PH-GFP) was therefore utilized to monitor PI3K activation. Akt-PH recognizes the PI3K product phosphatidylinositol-(3,4,5)-trisphosphate (PIP_3_) [and its hydrolytic product PI(3,4)P_2_], therefore this molecule tagged with fluorescent proteins gives a measure of PI3K activation with spatiotemporal precision by its translocation to specific subcellular organelles. Because a dominant negative effect of the expression of this protein on influenza infection has been reported [13], we carefully identified an expression level of PH-GFP that would avoid inhibition of infection (Figure S3A, B). Under this condition, accumulation of PH-GFP reflecting PI3K activation promoted by influenza virus infection could be observed not only at the plasma membrane but also in vesicular structures, in which influenza virus particles and Rab5 were colocalized (Figure 6A). Given that expression of Ras S17N, as well as LY294002 treatment, inhibited this colocalization, we can say that PIP_3_ accumulation in the early endosome requires Ras in addition to PI3K activity *per se*. Interestingly, whereas virus particles could localize in both early (Rab5 positive) and late (Rab7 positive) endosomes, the fluorescent protein-tagged PH domain could do so only in early endosomes (Figure 6A, B). These findings suggest that PI3K is also required for the transition from early endosomes to late endosomes where PI3K activity is already diminished; inhibition of PI3K activity actually resulted in the retention of viral particles in early endosomes (see Figure S2C). Furthermore, upon viral infection, Ras colocalized with viral particles, the PH domain, and Rab5 in a manner dependent on Ras and PI3K activities (Figure 6C). Given that Ras is indispensable for activating PI3K in the endosomes [19], these results together underscore an important role for the Ras–PI3K pathway in PIP_3_ production in early endosomes upon viral infection.

## Discussion

The PI3K pathway has gained general acceptance as being one of the key regulators for the cellular entry of a range of viruses and intracellular bacteria [13], [14], [28], [29], [30]; however, the mechanistic scenario in which PI3K functions has been far from understood. We hereby show that Ras-mediated PI3K activation is mainly required for virus entry during infection. This is consistent with reports that PI3K inhibition is effective in reducing titers of progeny viruses only when the inhibitor is applied earlier than 4 h.p.i., whereas the treatment at 4 h.p.i. or later is not able to display this effectiveness anymore [13], [14]. Given that lack of Ras–PI3K binding failed to localize PI3K in the endosomes (Figure S1A, B), Ras plays a determining role in the virus-induced, transient activation of PI3K in the endosomes. Notwithstanding this PI3K activation, phosphorylation of the major PI3K downstream molecule Akt at this early stage was not significant, and became prominent only at post-entry stages (Figure 5B). These kinetics of Akt phosphorylation are consistent with a previous report, where transient Akt phosphorylation is detected with lesser amplitude than that observed at a late stage, in a manner specific on cell contexts and viral strain [13]. These observations regarding the small amplitude of Akt phosphorylation may be accounted for by the restricted activation of PI3K in early endosomes, as we presented here, and the proportion of Akt activated by Ras-PI3K to total Akt can determine the extent of phosphorylated Akt detected by immunoblotting. Alternatively, given that the expression of wild-type or dominant-negative forms of Akt does not significantly alter virus entry [13], [14], PI3K in the endosomes may utilize PIP_3_ binding factors other than Akt to regulate virus entry.

On the other hand, our findings do not rule out the implication of PI3K-Akt signaling in late events of infection such as replication. In fact, prominent Akt phosphorylation was observed at 6 h.p.i. by immunoblotting (Figure 5A, B), as reported previously [13], [14]. Because the time course of this Akt activation is apparently different from that of Ras activation, slow Akt activation may be independent of Ras, but rather mediated by the viral protein NS1 through direct binding to the PI3K regulatory subunit p85β [8], [9], [10], [11], [12]. The PI3K–Akt pathway is also reported to participate in events associated with late steps, including the regulation of interferon production through interferon regulatory factor (IRF)-3 phosphorylation in response to double-stranded RNA [13], [31], [32].

How influenza viruses upregulate Ras–PI3K signaling at an early stage for their efficient entry remains obscure. The first step of influenza virus infection is adsorption to sialylated proteins on the cell surface. Thus, the type of sialylation of cell surface proteins is of great importance to determine virus host specificity; however, none of the sialylated proteins has been identified as a single specific receptor for this virus to date. On the other hand, it has recently been reported that the mannose 6-phosphate receptor is a sialic acid-independent receptor for influenza viruses in macrophages [33]. Given that the PR8 strain, used in this study and capable to activate Ras, cannot bind to the macrophage cell surface molecules, including this receptor, after sialidase treatment [33], signal transducers from influenza virus to Ras should be sialylated proteins. In this regard, it is noteworthy that the receptor tyrosine kinase fibroblast growth factor receptor is in a list of host factors implicated in influenza virus infection in several genome-wide studies [3], [4], [5], [6]. However, other acceptor mechanism(s) are likely to be dominant; our preliminary experiments show that the receptor inhibitor produced marginal effects on the infection and the level of phosphorylated tyrosine in the total cell lysate was not strikingly altered by the infection (data not shown).

In the present study, we utilized PI3Kγ because this class IB PI3K can be expressed in a manner independent of the regulatory subunits [34], [35], which allows for the complementary expression of the catalytic subunit in the *Pik3cg*-deficient cells, as we demonstrated. One may raise a concern about the use of fibroblasts as a model because the expression pattern of this molecule has been thought to be restricted within leukocytes. However, recent accumulating evidence indicates that PI3Kγ functions and is expressed in a much wider range of cell contexts than previously believed; e.g. endothelial cells, cardiomyocytes, cardiofibroblasts, and a vast majority of solid cancer cells [36], [37], [38], [39]. In the present study, the genetic ablation in fibroblasts indeed resulted in a decreased endocytic ability and influenza infectivity (Figures 1–3). Moreover, a similar inhibitory effect on these phenomena was also observed by the PI3Kγ-specific inhibitor AS605240 in several cell lines tested (data not shown), proving that PI3Kγ is apparently indispensable for operating influenza virus entry in a variety of cell lines including fibroblasts. Other subtypes of PI3K including class IA (PI3Kα and β) may also participate in influenza virus infection because the pan-PI3K inhibitor LY294002 achieved more efficient inhibitions than pharmacologic and genetic inhibition of PI3Kγ.

Among the stages of the influenza life cycle, viral entry is one of the remaining sanctuaries to be targeted for prevention and therapy. In addition, virus internalization after adsorption is a relatively non-specific process and is conserved over a range of viral subtypes and species. It therefore presents a promising target for achieving potent therapeutics with a broad spectrum of efficacy and a lower rate of resistance. To this end, a number of studies have attempted to identify the mechanism by which influenza enters cells and have implicated clathrin-mediated endocytosis, through which about 60% of the virus particles are indeed internalized [40], [41], [42]. However, expression of a dominant-negative form of Eps15 and knockdown of epsin 1, both of which are involved in clathrin-mediated endocytosis, do not hamper influenza virus infection [41], [43]. In contrast, ablation of the Ras–PI3K interaction restrained influenza virus internalization via inhibiting clathrin-independent endocytosis (Figures 1 and 3), underscoring Ras–PI3K-mediated, clathrin-independent endocytosis as one of the key pathways of influenza virus entry, whereas clathrin-dependent endocytosis is a supportive one. Our finding may also be applied to other viruses, apart from influenza viruses, namely the severe acute respiratory syndrome (SARS) coronavirus or dengue virus, both of which are known to infect via clathrin-independent endocytosis [44], [45].

In summary, the Ras–PI3K interaction, identified here as a host-oriented mechanism for influenza internalization, is implicated in very specific cellular phenomena, including clathrin-independent endocytosis and endosomal maturation, and is separate from clathrin-dependent endocytosis, which is necessary for a number of important biological events, such as receptor downregulation and low-density lipoprotein incorporation. In fact, adult mice deficient in Ras binding to PI3Ks are healthy [23]. Inhibiting this signaling pathway could lead to the development of a useful, well-tolerated prevention strategy against a variety of viral infections, which would aid in the development of effective countermeasures against viral pandemics.

## Materials and Methods

### Plasmids

cDNA for PI3Kγ was obtained from L. Stephen (the Babraham Institute, UK). PCR-mediated mutagenesis was performed to obtain cDNA encoding the K251E mutant, which harbors a mutation within the RBD that abrogates the interaction with Ras. The resulting PCR products were subcloned into the *Xho*I/*Not*I sites of pCAGGS-3×HA [46]. The expression vectors for GFP-Rab5 and GFP-Rab7 were kindly provided by T. Tsuboi (University of Tokyo, Tokyo, Japan), and those for the HA-tagged wild-type PI3Kγ, and CFP-tagged Akt-PH were described previously [19]. cDNAs for Rab5 and Rab7 were amplified by PCR and subcloned into the *Xho*I/*Not*I sites of pCAGGS-ECFP and pCAGGS-Venus vectors [47], respectively. Expression plasmids for wild type (WT) and the dominant negative mutant (S17N) of Ras were kindly provided by M. Matsuda (Kyoto University, Kyoto, Japan) [48]. Of note, these vectors are designed for Ras expression to be monitored by the fluorescence of bicistronically expressed GFP or RFP.

### Cells and transfection

Mouse embryonic fibroblasts (MEFs) deficient in *Pik3cg* were prepared as described [27] and maintained in DMEM (Sigma, St. Louis, MO) supplemented with 10% FBS at 37°C in a humidified atmosphere containing 5% CO_2_. Expression vectors for wild-type and mutant PI3Kγ were linearized by *Sca*I and introduced into the cells using nucleofection according to the manufacturer's recommendations (Amaxa Biosystems, Cologne, Germany). Starting at 2 days, the cells were cultured in DMEM containing 0.5 mg/ml G418 sulfate (Sigma), and the resistant colonies were collectively isolated. Expression of each protein was confirmed by immunoblotting (see Figure S1C). Stably transfected cells were maintained in DMEM supplemented with 0.2 mg/ml G418. Human embryonic kidney 293T (CRL-11268) and Cos-1 cells (JCRB9082) were obtained from American Type Culture Collection (Manassas, VA) and the Japanese Collection of Research Bioresources cell bank, respectively, and maintained in DMEM supplemented with 10% FBS, and MDCK cells (JCRB9029) were maintained in MEM supplemented with 10% FBS. For transfection, expression plasmids were introduced with FuGene HD (Roche, Basel, Switzerland) according to the manufacturer's recommendations.

### Reagents and antibodies

AlexaFluor488- or AlexaFluor546-labeled dextran (*M*r 10×10^3^), AlexaFluor546-labeled transferrin, LysoTracker Red, AlexaFluor594-conjugated anti-mouse antibody, and AlexaFluor647-conjugated anti-rat or anti-mouse antibody were purchased from Invitrogen (Carlsbad, CA). LY294002 was obtained from Calbiochem (San Diego, CA). An antibody to EEA-1 was purchased from BD Bioscience (San Jose, CA), that to HA was from Roche, that to Ras was from Oncogene Research Product (Cambridge, MA), that for β-actin was from Santa Cruz Biotechnology (Santa Cruz, CA), and those to phospho-Akt and Akt were from Cell Signaling Technology (Danvers, MA). The anti-NP antibody was a kind gift from A. Takada (Hokkaido University, Sapporo, Japan).

### Viruses

Influenza virus strains A/Puerto Rico/8/34 (H1N1; PR8) and A/Aichi/2/68 (H3N2; Aichi) were propagated in the chorioallantoic cavity of 10-day-old embryonated hen eggs for 48 hours at 37°C. For infection, MEFs were washed with PBS and infected with PR8 or Aichi at a multiplicity of infection (MOI) of 250 for 1 hour at 37°C. The inoculum was aspirated, and the cells were incubated with DMEM for 2 days. The amount of infectious virus in the cell supernatants was determined using common plaque assays as described previously [49], [50]. Briefly, confluent monolayers of MDCK cells in 12-well plates were inoculated with the supernatants. After 1 hour of adsorption, the cell supernatants were removed, and the cells were overlaid with MEM containing 1% Bact-agar and trypsin (5 µg/ml). Plaques were enumerated after incubation at 35°C and 5% CO_2_ for 2 days.

### Immunofluorescence and live cell imaging

Cells were fixed in 3% paraformaldehyde for 15 minutes at RT, permeabilized with 0.1% Triton X-100 in PBS for 4 minutes at RT, and then incubated with 1% bovine serum albumin to block non-specific binding of antibodies. The cells were further incubated with primary antibodies at 4°C overnight, followed by AlexaFluor594- or AlexaFluor647-conjugated secondary antibodies (1∶200 dilution, Molecular Probes, Invitrogen) for 1 hour at RT in the dark. Images were acquired using an FV-1000 confocal microscope (Olympus, Tokyo, Japan).

To assess endocytosis, cells plated on a collagen-coated 35 mm diameter glass base dish (Asahi Techno Glass Co. Tokyo, Japan) were incubated with 500 µg/ml fluorescence-conjugated dextran or transferrin for 30 minutes, washed extensively with PBS, and transferred into phenol red-free DMEM/F12 (Invitrogen). The cells were then imaged using an Olympus IX-71 microscope equipped with a CoolSNAP HQ cooled charge-coupled device (Photometrics, Tucson, AZ). Visualized vesicles were extracted using the “Top Hat” function of the MetaMorph image processing software (Universal Imaging, Downingtown, PA), followed by quantification of their fluorescence intensities. For the analysis of endosome maturation, cells were simultaneously labeled with AlexaFluor488-dextran and 100 nM LysoTracker Red for 10 minutes. After washing with PBS, the cells were further incubated with LysoTracker Red, followed by quantification as described above.

### Immunoblotting and Ras activation assay

Cells were lysed in RIPA buffer (50 mM Tris–HCl pH 7.4, 150 mM NaCl, 1 mM EDTA, 1% NP-40, 0.1% SDS, 0.5% sodium deoxycholate, and 1 mM Na_3_VO_4_) for 20 minutes on ice, and the lysate was clarified by centrifugation. The supernatants were subjected to SDS–PAGE, and separated proteins were blotted on polyvinylidene difluoride membranes (Bio-Rad, Hercules, CA). Signals were developed by ECL Western Blotting Detection Reagent (GE Healthcare, Little Chalfont, UK) and detected using an LAS-1000UV mini image analyzer (FUJIFILM, Tokyo, Japan).

*Escherichia coli* expression vectors for the GST-fused RBD of c-Raf1, pGEX-Raf-RBD, were obtained from S. Hattori (Kitasato University, Tokyo, Japan). Detection of GTP-bound Ras was performed essentially as described previously [26] with slight modifications [47]. Briefly, cells were lysed in lysis buffer (50 mM Tris–HCl pH 7.4, 150 mM NaCl, 5 mM MgCl_2_, 1% NP-40, 0.5% sodium deoxycholate, 0.1% SDS, and 1 mM Na_3_VO_4_), clarified by centrifugation, and incubated with GST-Raf-RBD for 30 minutes. The resulting complexes of GTP-bound Ras and GST-Raf-RBD were precipitated with glutathione–Sepharose beads, washed twice with lysis buffer, and eluted. GTP-bound Ras was detected by immunoblotting.

### Statistics

All data, unless otherwise specified, are expressed as box and whisker plots, where the highest and lowest boundaries of the box represent the 25th and 75th percentiles, respectively, and whiskers above and below the box designate the 5th and 95th percentiles, respectively; the line within the box indicates the median value. Alternatively, data are displayed in some figures as the mean ± s.d. of three independent experiments performed in triplicate. Data were subjected to one-way analysis of variance, followed by comparison using a Student's *t*-test to evaluate the differences. *P* values obtained from the test are found in the figure legends.

## Supporting Information

Figure S1 **Establishment of cell lines stably expressing wild-type or mutant PI3K that lack the ability to bind Ras.** (A and B) The K251E mutant failed to translocate to the endosomes by active Ras. Cells expressing wild-type PI3Kγ or its K251E mutant along with GFP-H-Ras G12V were fixed and incubated with anti-HA and anti-EEA-1 antibodies, followed by further incubation with a mixture of secondary antibodies. The cells were then observed by confocal microscopy. The right panels show whole-cell images to indicate where the left panels are from (A). Fluorescence intensities of AlexaFluor594 (Alexa594, blue), GFP (green) and AlexaFluor647 (Alexa647, red), along with the line in the merged image in (A), were plotted from *a* to *b*. Note that overlapping peaks indicate colocalization (B). This indicates that the K251E mutant of PI3Kγ cannot localize to the endosomes by active Ras. (C) Total cell lysates obtained from each cell line denoted at the top were subjected to SDS-PAGE, followed by immunoblotting with an anti-HA antibody. Expression levels of β-actin were used as a loading control. The data shown are representative of three independent experiments. (D) Impairment of endosome maturation in cells lacking Ras–PI3K binding. Cells processed as in Figure 1C were observed using a confocal microscope. Representative images are shown.
(EPS)

Figure S2 **Delayed virus trafficking in the absence of the Ras–PI3K interaction.** (A and B) Cells processed as in Figure 3C, D were observed by confocal microscopy. (A) Total virus particles visualized by an anti-NP antibody were plotted. Note that the total number of virus particles, including adsorbed viruses and incorporated viruses, is comparable throughout the experimental conditions. (B) Representative images are shown. Fluorescence intensities of CFP (blue), YFP (green) and AlexaFluor594 (Alexa594, red), along with the line in the merged image, were plotted from *a* to *b*. Note that overlapping peaks indicate colocalization. Yellow arrowheads indicate colocalization between NP and Rab7, and purple arrowheads indicate colocalization between NP and Rab5. The latter indicates delayed trafficking of virus particles. (C) Relative fluorescence intensities of NP colocalized with Rab5 (▪) and Rab7 (□) are plotted.
(EPS)

Figure S3 **Optimization of the expression level of Akt-PH to analyze PIP3 production induced by influenza infection.** (A) MEFs expressing Akt-PH-GFP were infected with PR8, stained with an anti-NP antibody, and observed by fluorescence microscopy. The arrowhead indicates a cell overexpressing Akt-PH in which influenza virus infection was inhibited. (B) Cells were categorized into groups, indicated at the bottom, by the fluorescence intensity of GFP. The fluorescence intensity of AlexaFluor594 (NP) for each cell is plotted. Notably, virus infectivity was comparable in cells with GFP fluorescence intensities less than 150 and in cells lacking expression of Akt-PH. On the basis of this result, we used cells satisfying this criterion to analyze PIP_3_ production induced by influenza virus infection.
(EPS)
